# BRAZILIAN CONSENSUS ON INCIDENTAL GALLBLADDER CARCINOMA

**DOI:** 10.1590/0102-672020190001e1496

**Published:** 2020-07-08

**Authors:** Grupo Internacional de Estudos de Câncer Hepatopancreatobiliar - ISG-HPB-Cancer, Felipe Jose F COIMBRA, Orlando Jorge M TORRES, Ruslan ALIKHANOV, Anil AGARWAL, Patrick PESSAUX, Eduardo de Souza M FERNANDES, Claudemiro QUIREZE-JUNIOR, Raphael Leonardo C ARAUJO, André Luis GODOY, Fabio Luis WAECHTER, Alexandre Prado de RESENDE, Marcio Fernando BOFF, Gustavo Rego COELHO, Marcelo Bruno de REZENDE, Marcelo Moura LINHARES, Marcos BELOTTO, Jose Maria A MORAES-JUNIOR, Paulo Cezar G AMARAL, Rinaldo Danesi PINTO, Tercio GENZINI, Agnaldo Soares LIMA, Heber Salvador C RIBEIRO, Eduardo José RAMOS, Marciano ANGHINONI, Lucio Lucas PEREIRA, Marcelo ENNE, Adriano SAMPAIO, André Luis MONTAGNINI, Alessandro DINIZ, Victor Hugo Fonseca de JESUS, Bhawna SIROHI, Shailesh V SHRIKHANDE, Renata D`Alpino PEIXOTO, Antonio Nocchi KALIL, Nicolas JARUFE, Martin SMITH, Paulo HERMAN

**Affiliations:** 1 Department of Gastrointestinal Surgery, AC Camargo Cancer Center, São Paulo, Brazil; 2 Department of Hepatopancreatobiliary Surgery, Federal University of Maranhão, São Luis, Brazil; 3 Department of Hepatopancreatobiliary Surgery, Moscow Clinical Scientific Center, Moscow, Russia; 4 Department of Gastrointestinal Surgery, Govind Ballabh Pant Hospital, New Delhi, India; 5 Department of Hepatopancreatobiliary Surgery, Nouvel Hopital Civil, University Hospital of Strasbourg, Strasbourg, France; 6 Department of Hepatopancreatobiliary and Transplant Surgery, Federal University of Rio de Janeiro, Rio de Janeiro, Brazil; 7 Department of Gastrointestinal Surgery, Federal University of Goiás, Goiânia, Brazil; 8 Department of Hepatobiliary Surgery, Federal University of São Paulo, São Paulo, Brazil; 9 Department of Gastrointestinal Surgery, Santa Casa de Porto Alegre, Porto Alegre, Brazil; 10 Department of General Surgery, Mater Dei Hospital, Belo Horizonte, Brazil; 11 Department of Oncology Surgery, Mãe de Deus Hospital, Porto Alegre, Brazil; 12 Department of Hepatopancreatobiliary Surgery, Hospital Walter Cantidio, Fortaleza, Brazil; 13 Department of Hepatopancreatobiliary Surgery, Hospital Albert Einstein, São Paulo, Brazil; 14 Department of Gastrointestinal Surgery, Santa Casa de São Paulo, São Paulo, Brazil; 15 Department of Gastrointestinal Surgery, Hospital São Rafael, Salvador, Brazil; 16 Department of Gastrointestinal Surgery, Hospital Santa Catarina, Blumenal, Brazil; 17 Department of Hepatopancreatobiliary Surgery, Hospital Beneficiência Portuguesa, São Paulo, Brazil; 18 Department of Hepatopancreatobiliary Surgery, Santa Casa de Belo Horizonte, Brazil; 19 Department of Hepatopancreatobiliary Surgery, Hospital NS das Graças, Curitiba, Brazil; 20 Department of Oncology Surgery, Hospital São Vicente, Curitiba, Brazil; 21 Department of Gastrointestinal Surgery, Hospital Sírio-Libanês, Brasilia, Brazil; 22 Department of Hepatopancreatobiliary Surgery, Ipanema Hospital, Rio de Janeiro, Brazil; 23 Department of Gastrointestinal Surgery, Santo Amaro University, São Paulo, Brazil; 24 Department of Hepatopancreatobiliary Surgery, São Paulo Medical School, São Paulo, Brazil; 25 Department of Gastrointestinal Oncology, AC Camargo Cancer Center, São Paulo, Brazil; 26 Department of Hepatopancreatobiliary and Oncology Surgery, Tata Memorial Hospital, Mumbai, India; 27 Department of Gastrointestinal Oncology, Hospital Alemão Oswaldo Cruz, São Paulo, Brazil; 28 Department of Gastrointestinal Oncology, Santa Casa de Porto Alegre, Porto Alegre, Brazil; 29 Department of Hepatopancreatobiliary Surgery, Universidade Católica, Santiago, Chile; 30 Department of Hepatopancreatobiliary Surgery, Chris Hani Baragwanath Academic Hospital, Johannesburg, South Africa

**Keywords:** Gallbladder, Cancer, Gallbladder cancer, Incidental gallbladder, Consensus, Vesícula biliar, Câncer, Câncer da vesícula biliar, Carcinoma incidental, Consenso

## Abstract

**Background:**

Incidental gallbladder cancer is defined as a cancer discovered by histological examination after cholecystectomy. It is a potentially curable disease. However, some questions related to their management remain controversial and a defined strategy is associated with better prognosis.

**Aim:**

To develop the first evidence-based consensus for management of patients with incidental gallbladder cancer in Brazil.

**Methods:**

Sixteen questions were selected, and 36 Brazilian and International members were included to the answer them. The statements were based on current evident literature. The final report was sent to the members of the panel for agreement assessment.

**Results:**

Intraoperative evaluation of the specimen, use of retrieval bags and routine histopathology is recommended. Complete preoperative evaluation is necessary and the reoperation should be performed once final staging is available. Evaluation of the cystic duct margin and routine 16b1 lymph node biopsy is recommended. Chemotherapy should be considered and chemoradiation therapy if microscopically positive surgical margins. Port site should be resected exceptionally. Staging laparoscopy before reoperation is recommended, but minimally invasive radical approach only in specialized minimally invasive hepatopancreatobiliary centers. The extent of liver resection is acceptable if R0 resection is achieved. Standard lymph node dissection is required for T2 tumors and above, but common bile duct resection is not recommended routinely.

**Conclusions:**

It was possible to prepare safe recommendations as guidance for incidental gallbladder carcinoma, addressing the most frequent topics of everyday work of digestive and general surgeons.

## RESUMO

**Racional:**

Carcinoma incidental da vesícula biliar é definido como uma neoplasia descoberta por exame histológico após colecistectomia videolaparoscópica. É potencialmente uma doença curável. Entretanto algumas questões relacionadas ao seu manuseio permanecem controversas e uma estratégia definida está associada com melhor prognóstico.

**Objetivo:**

Desenvolver o primeiro consenso baseado em evidências para o manuseio de pacientes com carcinoma incidental da vesícula biliar no Brasil.

**Métodos:**

Dezesseis questões foram selecionadas e para responder as questões e 36 membros das sociedades brasileiras e internacionais foram incluídos. As recomendações foram baseadas em evidências da literatura atual. Um relatório final foi enviado para os membros do painel para avaliação de concordância.

**Resultados:**

Avaliação intraoperatória da peça cirúrgica, uso de bolsas para retirar a peça cirúrgica e exame histopatológico de rotina, foram recomendados. Avaliação pré-operatória completa é necessária e deve ser realizada assim que o estadiamento final esteja disponível. Avaliação da margem do ducto cístico e biópsia de rotina do linfonodo 16b1 são recomendadas. Quimioterapia deve ser considerada e quimioradioterapia indicada se a margem cirúrgica microscópica seja positiva. Os portais devem ser ressecados excepcionalmente. O estadiamento laparoscópico antes da operação é recomendado, mas o tratamento radical por abordagem minimamente invasiva deve ser realizado apenas em centros especializados em cirurgia hepatopancreatobiliar minimamente invasiva. A extensão da ressecção hepática é aceitável até que seja alcançada a ressecção R0. A linfadenectomia padrão é indicada para tumores iguais ou superiores a T2, mas a ressecção da via biliar não é recomendada de rotina.

**Conclusões:**

Recomendações seguras foram preparadas para carcinoma incidental da vesícula biliar, destacando os mais frequentes tópicos do trabalho diário do cirurgião do aparelho digestivo e hepatopancreatobiliar.

## INTRODUCTION

Gallbladder cancer (GBC) is a rare malignancy associated with a dismal prognosis with over one-third of patients presenting with distant metastasis at the time of diagnosis. Chile, Japan and Northern India are areas of high incidence and significant mortality. Due to the aggressive nature of gallbladder cancer, five-year survival rates ranges between 5% to 15%.^1^ ^,^ ^42^ The disease can be suspected preoperatively, identified intraoperatively or at the examination of the removed gallbladder, or incidentally found after histology report. Incidental gallbladder carcinoma (IGBC) is defined as a cancer discovered at the histological examination of the specimen after cholecystectomy (index cholecystectomy, IC) as early gallbladder cancers does not have specific symptoms. IGBC represents approximately 70% of the gallbladder cancers in non-endemic areas and occur between 0.2% and 3% of patients undergoing cholecystectomy. IGBC is a potentially curable disease and a better prognosis is associated with the adoption of an adequate surgical strategy^1^ ^,^ ^42^. Overall, patients with incidental gallbladder cancer have significantly better median survival (26.5 months) than patients with non-incidental or primary gallbladder cancer (9.2 months). The management after the diagnosis of IGBC is crucial for the prognosis and is a challenging issue due to the absence of established guidelines. IGBC may pose several dilemmas for further management and the impact on outcome. Discussion at a multidisciplinary team meeting has become more common in recent years and the recommendation is decided thereafter^1^ ^,^ ^42^.

The aim of this study was to create a consensus guideline for management of patients with incidental gallbladder carcinoma.

## METHODS

The International Study Group of Hepatopancreatobiliary Cancer (ISG-HPB-Cancer) Committee appointed a Brazilian chair to prepare the national guideline development, and selected 36 respondents on the basis of their experience in gastrointestinal cancer. Members of the Brazilian College of Hepatopancreatobiliary Surgery, from the Brazilian Society of Surgical Oncology, and the Brazilian College of Digestive Surgery were contacted by email to make their contributions to the guidelines. Members of the Brazilian Society of Clinical Oncology were also included and members of International Hepatopancreatobiliary Association (IHPBA) with expertise in gallbladder carcinoma were invited to join the panel as international experts. Consensus was based on Delphi questionnaire, which was initially devised by the Brazilian experts members and approved by the ISG-HPB-Cancer.

The Brazilian members of the panel extracted specific questions related to incidental gallbladder carcinoma from the literature by a PubMed search. At first, 19 questions were selected, and after discussion 16 questions were included in the consensus by modified Delphi approach. In the first round the members of the panel were divided into three groups and groups I and II had to answer 5 (1-5 and 6-10 respectively) questions each, based on current evidence. Group III had to answer six questions (11-16). At least two international experts from India, Chile, South Africa, France and Russia were included in each group. A total of seven worldwide experts were invited to achieve international representation.

After receiving the answers, the second-round consisted of sending the answers to a different group (group I received the answers from group II and III; group II received the answers from groups I and III; and group III received answers from group I and II). A critical review of all answers was performed by the panel and sent to the committee. In the third round three Brazilian experts analyzed the rational and the statements derived from the 16 questions and sent to the panel of international experts for evaluation. The statements were based on the available evidence and Brazilian and international experts’ opinions during the fourth round. To establish a consensus, on the fifth round, the statements were sent to the members of the panel for agreement. The percentage of agreement was included at the end of each statement. As previously defined by the experts, a modified Delphi consensus was reached when at least 80% of the panel agreed with the final statement. 

## RESULTS

All the 16 questions had more than 80% of agreement and individual statements with the percentage of agreement for each question have been proposed.

### Question 1 All cholecystectomy specimens should be opened and examined intraoperatively?

Gallbladder cancer is an uncommon disease and the overall 5-year survival rate is currently reported to be 5% to 15%, with a mean overall survival of 3-13 months. Although primary gallbladder cancer remains common in endemic regions, in non-endemic regions approximately 55% to 70% are found incidentally during or after an elective cholecystectomy for gallstones. Incidental gallbladder carcinoma represents 0.19% to 2.3% of all patients undergoing laparoscopic or open cholecystectomy. In these cases, the 5-year overall survival can reach 99% for T1aN0 and 70% for T2N0 cancer cases. In only 30% of patients with gallbladder cancer related to cholecystectomy the disease is suspected preoperatively. Several risk factors have been identified for incidental gallbladder carcinoma, and the possibility of incidental gallbladder carcinoma in patients without risk factors is very low. The most common risk factors for GBC are: age ≥65 years old, previous or presentation with cholecystitis, jaundice, women, raised alkaline phosphatase, focal gallbladder wall thickening ≥5 mm, biliopancreatic maljunction and a dilated bile duct^32^ ^,^ ^42^. Even in the absence of these risk factors examination of the gallbladder specimen should be considered as it is a simple procedure, can potentially detect suspicious lesions and does not adversely influence histopathological examination of the specimen^32^ ^,^ ^42^.

#### Consensus statement

*Most of GBC are diagnosed incidentally and all cholecystectomy specimens should be opened and examined by the surgeon intraoperatively especially when risk factors are present. Frozen section should be performed in all suspected cases. All specimens must be sent for histopathological evaluation in order not to miss incidental gallbladder cancer. (Agreement 91.4%)*

### Question 2 Use of a retrieval bag is mandatory in all laparoscopic cholecystectomies?

At present, the gallbladder is removed by laparoscopic cholecystectomy in more than 70% of cases. Port-site metastases following laparoscopic cholecystectomy for unsuspected gallbladder cancer were initially reported in 1991. Implantation of tumor cells at port sites can occur by both direct (mechanical factors) and indirect mechanisms (pneumoperitoneum spillage). Experimental studies and clinical evidence from other laparoscopic oncological procedures like colorectal surgery where port-site recurrence has been reduced to 1% with the use of appropriate preventive measures suggest that mechanical factors (gallbladder perforation with bile spillage and poor specimen extraction techniques) play an important role in port site metastases rather than pneumoperitoneum. The specimen might accidentally open during retrieval. Therefore the use of retrieval bags is important for preventing port site contamination, besides enabling easy handling of the resected specimen. Even if malignancy is not suspected preoperatively, as in incidental gallbladder carcinomas, the use of a retrieval bag minimizes the risk of tumor cell dissemination in and around the incision tract^21^ ^,^ ^42^.

#### Consensus statement

*Routine use of retrieval bags is highly recommended because it is not always possible to foresee problems with the gallbladder retraction, and it may often be too late to use a bag when gallbladder rupture occurs before or during the extraction through a small incision. With the availability of retrieval bags that are easy to handle and relatively non-expensive, it is now recommended to use bags routinely in laparoscopic cholecystectomies. It is very important to avoid intraoperative gallbladder perforation (lower monopolar energy intensity and meticulous dissection) when a suspicious lesion is intraoperatively detected. (Agreement 91.4%)*

### Question 3 Routine histopathology of the gallbladder to detect inapparent gallbladder cancer is mandatory?

The early-stage gallbladder carcinoma in which surgical resection provides the greatest benefit is difficult to detect preoperatively and is often missed even after intraoperative examination of the cholecystectomy specimen. Moreover, these patients presented early and achieved a better R0 resection rate and better overall survival, compared to those in whom the gallbladder was not sent for histopathology, when recurrence occurs, a poor resectability rate and poor long-term survival are observed^2^ ^,^ ^15^ ^,^ ^29^ ^,^ ^33^.

It has been standard practice to submit all gallbladders removed for gallstone disease to routine histopathology to exclude gallbladder malignancy. In recent years; however, some authors have questioned the role of routine histopathology of cholecystectomy specimens. These authors support selective approach by claiming that gallbladder carcinoma is unlikely to occur in normal looking gallbladder and absence of risk factors. Tumors undetectable during macroscopic evaluation of the gallbladder are usually early stage tumors (Tis, T1a) where simple cholecystectomy might be enough. Hence, routine rather than selective histopathological evaluation have been advocated by these groups. However, incidental gallbladder cancer has been reported even in patients with normal findings on macroscopic examination of the cholecystectomy specimen and authors who are for routine evaluation agree that early stage tumors can easily be overlooked in macroscopic specimen examination^2^ ^,^ ^15^ ^,^ ^29^ ^,^ ^33^.

#### Consensus statement

*Routine histopathology is recommended for all gallbladder specimens. Patients in whom a cholecystectomy specimen was sent for routine histopathology, all incidental gallbladder cancers are expected to be found. The pathological analysis must include at least three sectors of the gallbladder and the cystic margin. In case of cancer finding, the histopathology report should inform the depth of invasion, margins, if the tumor is located on the hepatic or peritoneal side and Rokitansky-Aschoff sinuses involvement. When using a selective approach of histopathological examination of cholecystectomy specimens, it is important to make a meticulous on-table evaluation of the specimen and to take into account the risk factors associated with gallbladder cancer. (Agreement 100 %)*

### Question 4 After index cholecystectomy and confirmed histopathology, how to evaluate the patient preoperatively?

Extent of preoperative evaluation is determined by the risk factors for metastatic disease in a given patient. The risk of metastasis is determined by preoperative, intraoperative and postoperative factors after index cholecystectomy. Preoperative factors include presenting symptom (pain or jaundice), extent of preoperative investigations before cholecystectomy, whether it is a true incidental gallbladder cancer or findings suspicious of malignancy missed during preoperative or intraoperative period, type of surgery (laparoscopy or open, or laparoscopy converted to open), emergency or elective surgery, intraoperative events like gallbladder perforation with bile spillage during dissection and the use of a retrieval bag to remove the specimen. It is also important to define if the tumor was located on the gallbladder liver bed or at the gallbladder peritoneal side, and the location of the tumor in the gallbladder itself (fundus, body or neck). The time interval from the first operation to the evaluation at a hepatobiliary center and T stage of the tumor is also vital*.* Chest computed tomography (CT) and abdominal CT or abdominal magnetic resonance imaging (MRI) should be performed to exclude disseminated disease. In patients with adverse risk factors as mentioned before PET-CT may be considered for preoperative staging. PET-CT has also a role for ruling out local residual disease and distant metastases^9,11, 19,24^. 

#### Consensus statement

*Information about the type of surgery performed, gallbladder perforation, bile spillage and the use of retrieval bag are essentials for identifying the risk of peritoneal carcinomatosis and define prognosis. Histopathological report and complementary evaluation confirming T stage may also be necessary to define for observation or reoperation. Chest and abdominal CT or abdominal magnetic resonance imaging (MRI) should be performed to exclude disseminated disease. In patients with concomitant liver steatosis or cirrhosis, the use of MRI is recommended. CT is preferred in patients with risk factors for metastatic disease. (Agreement 100 %)*

### Question 5 Which is the ideal timeframe between cholecystectomy and radical surgery for incidentally discovered gallbladder cancer?

Current guidelines for management of incidental gallbladder cancer recommend re-resection for T1b, T2, and T3 lesions, unless contraindicated by advanced disease or poor performance status. Re-resection may also be considered in patients with T1a disease if the histopathological report is from an unreliable center or the paraffin blocks are not available for review. Reports of recurrence after T1a disease are usually due to misinterpretation of T stage based on a few sections in the literature. There are few data on the timing of re-resection, following the initial cholecystectomy. Reoperation should be performed as early as possible once final histopathological staging is available, metastatic workup is complete and a patient is fit for reoperation. Preliminary results based on frozen section analysis can be difficult to interpret and may be unreliable in the setting of acute inflammation. Reoperation too late (after eight weeks) may allow too much time for disease dissemination. The stage of the disease, tumor biology, and technical considerations, plays an important role in defining the optimal timing of reoperation. Studies have shown that prolonged interval between index cholecystectomy and completion radical cholecystectomy is an adverse prognostic factor. However, the stage of the disease is more important than the time interval between index cholecystectomy and re-resection as prognostic factor for recurrence^7^ ^,^ ^13^ ^,^ ^42^.

#### Consensus statement

*The choice of timing for reoperation is largely dictated by the inflammatory process of the first procedure. Waiting time is necessary in order to minimize complications and maximize patient safety. Reoperation should be performed as early as possible once final histopathological staging is available, metastatic workup is complete and patients is fit for reoperation which may take 2-4 weeks after index cholecystectomy depending on the time of referral and the stage of the disease. Prolonged interval between index cholecystectomy and completion of radical cholecystectomy adversely affects overall outcome.* Radical reoperation is recommended for patients with disease ≥pT1b even if they present after two months of index cholecystectomy. *(Agreement 97.1%)*

### Question 6 What is the role of systemic chemotherapy in the management of incidental gallbladder cancer?

There is no current evidence for the use of chemotherapy in the neoadjuvant setting prior to re-resection. In patients with locoregionally advanced disease (i.e., nodal disease or evidence of other high-risk disease), neoadjuvant chemotherapy should be considered according to some panels and in study protocols^1^ ^,^ ^8^ ^,^ ^36^ ^,^ ^41^. Given the high risk of relapse following surgery, interest in adjuvant treatment has been high. In 2015 a meta-analysis of 10 retrospective studies involving 3,191 patients reported improvement in overall survival for patients with biliary tract cancer treated with adjuvant chemotherapy with greatest benefit in patients with non-curative surgical resection, lymph node-positive disease, and AJCC stage greater than 2. Recently reported, BILCAP trial was a phase III randomized controlled study with 447 patients (18% of them with muscle-invasive gallbladder cancer) conducted in the UK, which demonstrated an improvement in median overall survival (53 months vs. 36 months, p=0.028) and recurrence-free survival (25 months vs. 18 months, p=0.03) for patients treated with adjuvant capecitabine compared to observation alone. This regimen has now become the recommended standard of care for resected muscle-invasive gallbladder cancer, regardless of the method of diagnosis. Based on the significant results of the BILCAP trial, the Expert Panel recommends that capecitabine for a period of six months should be offered as adjuvant therapy to patients with resected gallbladder cancer. Despite higher doses used in the trial, Brazilian oncologists recommend the dose of 2,000 mg/m^2^ from D1 to D14 every 21 days. Patients with gallbladder cancer and a microscopically positive surgical margin resection (R1 resection) may be offered chemoradiation therap^1^ ^,^ ^8^ ^,^ ^36^ ^,^ ^41^.

#### Consensus statement

*The core treatment of gallbladder cancer incidentally found on postoperative histopathology is radical surgery. For histologically-proven T1a and T1b gallbladder cancers if the gallbladder was completely resected during the previous surgery or after re-resection no additional chemotherapy should be offered. There is low evidence for neoadjuvant chemotherapy in gallbladder cancer. In cases of T2 or above gallbladder cancers, and for patients with high risk of microscopically positive surgical margins, additional use of chemotherapy based on capecitabine for six months should be offered after extended cholecystectomy and lymphadenectomy.(Agreement 100 %)*

### Question 7 Staging laparoscopy before reoperation is recommended for all patients?

Laparoscopy has been shown to be an important tool in the management of patients with gastrointestinal malignancies. It provides the ability to identify disseminated disease and avoid unnecessary laparotomy. Some previous studies have reported the benefits of staging laparoscopy in preventing a non-therapeutic surgical exploration in 38% to 62% of patients with gallbladder carcinoma. Associated laparoscopy and intra-operative ultrasound is helpful in detecting metastases on liver surface, peritoneum and regional lymph nodes, obviating non-therapeutic laparotomy in up to 48% of cases. Agarwal et al. ^3^ reported that staging laparoscopy identified 94.1% of detectable lesions thereby avoiding a non-therapeutic laparotomy in 55.9% of patients with unresectable disease^3^ ^,^ ^10^ ^,^ ^20^. In patients with hepatobiliary cancers, the incidence of unresectable disease is high (25-75%). Staging laparoscopy is frequently utilized in order to decrease lengths of stay and to start palliative chemotherapy in patients not amenable to resection. As imaging technology improves, however, an increasingly larger proportion of patients are identified during preoperative staging examinations as having unresectable cancer. Butte et al^10^ showed that a positive cholecystectomy margin and poor tumor differentiation were independent factors associated with disseminated disease at re-exploration. The likelihood of disseminated disease is correlated with T-stage and was present in over one-quarter of patients with T3 tumors^3^ ^,^ ^10^ ^,^ ^20^. Intra-abdominal adhesions due to prior cholecystectomy might decrease the yield and accuracy of staging laparoscopy in incidental gallbladder cancer. Hence, two additional ports along the line of planned incision should be placed in addition to camera port to perform adhesiolysis^3^ ^,^ ^10^ ^,^ ^20^.

#### Consensus statement

*Staging laparoscopy before gallbladder cancer reoperation is recommended for patients with T1b and above gallbladder cancers. Potential of detecting metastases may be higher than for primary GBC due to delayed presentation and risk factors such as bile spillage due to gallbladder perforation, presence of positive margins and high-grade tumors. (Agreement 97.1%)*

### Question 8 Which is the best treatment for T1b tumors?

T1a gallbladder cancer is defined as cancer confined to the mucosa and T1b as cancer confined to the *muscularis mucosa*. Univariate analysis showed that depth of invasion (T1a vs T1b), histopathological tumor differentiation, and surgical margins (R0 vs R1/R2) were significant prognostic factors. For patients with a T1b tumor, multivariate analysis revealed that R1/R2 resection (p = 0.017) and lymph node metastasis (p = 0.001) significantly predicted a poor prognosis. In a systematic review of the T1 gallbladder cancer, lymph node metastasis was present in approximately 11% of all cases, and the recurrence rate was 9.3%. This rate of recurrence is higher in patients with T1b gallbladder cancer who had undergone a simple cholecystectomy. The 1-year survival drops down to 50% for T1b tumors not undergoing radical excision. Lee et al.^28^ observed that patients with T1b stage gallbladder carcinoma that underwent radical cholecystectomy presented a significant better prognosis when compared to those submitted to a simple cholecystectomy. However, in the international multicenter study by Kim et al, ^25^ simple cholecystectomy showed similar results of recurrence and survival to radical surgery in T1b tumors^44^ ^,^ ^45^. Yoon et al.^45^ showed that when lymph node metastasis occurs, radical cholecystectomy has a better prognosis when compared to cholecystectomy. The overall 5-year survival rate of the simple cholecystectomy and the extended cholecystectomy was 88.8 % and 93.3 %, respectively; this difference was not significant (p = 0.521). However, recurrence occurred in 11.1 % of patients, all in the simple cholecystectomy group ^25,28,44,45^.

#### Consensus statement

*There is a consensus that R0 resection represents the strongest prognostic factor forlong-term outcome and chance for cure in patients with gallbladder cancer. Radical cholecystectomy with lymphadenectomy should be recommended for patients with T1b gallbladder cancer who are not at increased risk of developing postoperative complications. (Agreement 97.1%)*

### Question 9 The cystic duct should be evaluated routinely?

Resection of the extrahepatic bile duct should not be performed routinely during extended radical resection. In recent studies it has been shown that the resection of the extrahepatic bile duct increases peri-operative morbidity and is not associated with an increase in long-term survival in patients with no positive cystic duct margin. Extrahepatic bile duct resection is useful as a standard operation for tumors involving (macroscopically or microscopically) the neck and/or the cystic duct of the gallbladder. Pawlik et al.^34^ reported that patients with positive cystic duct margin are significantly more likely to have residual/additional cancer at the common bile duct (42% vs. 4.3%).^34^ In patients with incidental gallbladder cancer a positive cystic duct margin is a strong and independent predictor of worse overall survival even if no further residual cancer is found. Therefore, the cystic duct margin status should be reported for each patient with incidental gallbladder cancer. A positive margin warrants prompt consideration for clearance of the cystic duct stump and extrahepatic bile duct resection to achieve negative margins and optimal oncologic outcome^34^ ^,^ ^38^ ^,^ ^41^.

### Consensus statement

*The determination of the cystic duct margin involvement is important for subsequent surgical decision making process, especially in gallbladder cancers located at the infundibulum. This procedure can help to determine the need for extended duct resection and should be evaluated routinely. Intra-operative frozen section of the cystic duct stump is mandatory in tumors located at the infundibulum and/or cystic duct.(Agreement 100 %)*

### Question 10 Is routine 16b1 lymph node biopsy in the management of incidental gallbladder cancer necessary?

In gallbladder cancer, involvement of the interaortocaval lymph node (16b1) represents an advanced disease with poor prognosis, equivalent to distant metastases. It represents between 19-38% of all patients with gallbladder cancer. Accurate preoperative evaluation is paramount in optimizing the management of patients with gallbladder cancer. Intraoperative biopsy and frozen-section analyses of these nodes have been proposed. ^5,31,42^However, the prognostic impact of these lymph node metastases is still under discussion and some series do not consider 16b1 lymph node metastasis as a contraindication for radical resection. Para-aortic lymph nodes involvement occurs in approximately 19% of patients with pT2-pT3 GB cancer. According to some studies, no significant difference on overall survival was evidenced among patients with or without metastatic para-aortic lymphatic involvement. The reason for variable survival outcomes reported in patients with positive 16b1 lymph node metastasis could be due to the differences in the pathway of lymphatic spread. A small group of patients with skip metastasis to 16b1 lymph nodes without significant lymphadenopathy in other stations might benefit from radical surgery. Preoperative endoscopic ultrasound guided FNAC of the 16b1 lymph nodes could detect metastatic spread and potentially avoid non curative resection in patients with extensive regional lymphadenopathy. Agarwal et al.^5^ observed that the incidence of positive lymph node 16b1 was higher in patients with locally advanced gallbladder cancer, jaundice and raised preoperative serum tumor marker levels. Consideration should be based whether or not R0 resection is possible^5^ ^,^ ^31^ ^,^ ^42^.

#### Consensus statement

*In gallbladder cancer, involvement of para-aortic lymph node (16b1) represents an advanced disease with poor prognostic and has been considered as metastatic disease (M1). Routine 16b1 lymph node biopsy in the management of incidental gallbladder cancer is recommended during gallbladder cancer reoperation for adequate staging and determination of oncological prognosis. Isolated 16b1 lymph node metastasis without significant regional lymphadenopathy or other adverse prognostic factors may not be a formal contraindication for radical surgery. (Agreement 94.2%)*

### Question 11 What is the adequate extension of liver resection? Gallbladder bed resection, hepatectomy of segments 4b and 5, or extended hepatectomy?

The depth of invasion through the gallbladder wall determines the standard surgical treatment for gallbladder cancer. Patients with pathological T2 (pT2) gallbladder cancer and no distant metastases should always be considered for radical resection. The prognosis of patients with pT2 gallbladder cancer treated with simple cholecystectomy is poor, and liver resection and regional lymphadenectomy are also necessary. Hepatectomy for pT2 gallbladder cancer is advisable because it provides an adequate tumor-free margin on the gallbladder bed^6^ ^,^ ^23^ ^,^ ^42^. The 5-year survival rate for pT1 cases is 85.9%, whereas the prognosis for pT3 and pT4 cases is 19.2% and 14.1%, at five years respectively. Liver resection is indicated because a tumor focus can be found up to 2 cm away from the margin of the primary tumor. In a study of the gallbladder vein drainage, the vein was found to perforate the gallbladder bed and to perfuse segment S4b and S5 in 37% and 52% respectively. However, a survey conducted in 2005 by the Japanese Society of Biliary Surgery including 293 patients with pT2 gallbladder cancers, revealed no difference in the survival rate between the gallbladder bed resection group and the S4b/5 resection group. In addition, they showed that the site of hepatic metastasis/recurrence was not confined to 4b/5 segments. In the present study they observed that lymph node metastasis could strongly affect the prognosis for gallbladder cancer patients. This study revealed that the extent of hepatectomy should not be considered as a prognostic factor as long as R0 resection is achieved^6^ ^,^ ^23^ ^,^ ^42^.

#### Consensus statement

*For Tis and T1a tumors no further resection is required. For T1b and higher stage tumors additional hepatic resection is indicated provided the patient is fit for surgery. Both gallbladder bed resection and segments IVb and V resection are an oncological acceptable procedure provided R0 resection is achieved. Extended hepatectomy is usually required in patients with locally advanced tumor with biliary and vascular involvement to achieve R0 margins. If R0 is achieved, major hepatectomies are not superior to non-anatomical resections of the gallbladder bed with part of segments IVb and V and is associated with higher morbidity. (Agreement 100 %)*

### Question 12 What is the optimal extent of lymph node dissection?

Oncologic extended resection remains the only effective and potentially curative treatment for gallbladder carcinoma. More than that, the most powerful predicting factor for survival is nodal status and worse survival is observed in node positive disease. Also, the involvement of regional lymph nodes in T2 tumors occurs in 19-62% and T3-T4 tumors 78-85%. N2 lymph nodes involvement occurs in 18-36% in T2 tumors and 42-71% in T3-T4 tumors. Thus, adequate lymph node staging should include intraoperative evaluation of suspicious regional nodes, and assessment of the aortocaval nodal basin. The cystic and pericholedochal nodes are the most commonly involved nodes. Patients with confirmed aortocaval nodal disease may not benefit from radical resection. For T2-T4 disease, the retrieval of at least six lymph nodes is recommended and includes N1 (cystic, pericholedochal, hilar nodes, hepatoduodenal ligament), and N2 (peripancreatic, periportal, peridutal, and common hepatic artery nodes). Provided that the quality of lymphadenectomy and the tumor biology have been demonstrated as important prognostic factors, the lymph node ratio has raised as an important predictor of survival after surgery. In this case, the dissection beyond the portal lymph nodes may be considered^1^ ^,^ ^26^ ^,^ ^39^ ^,^ ^40^ ^,^ ^43^.

#### Consensus statement

*For T2-T4 disease, “standard” lymph node dissection requires the retrieval of at least six lymph nodes and includes N1 (cystic 12c, pericholedochal 12b, hilar nodes 12h, proper hepatic artery node 12a), and N2 (peripancreatic 13a, periportal 12p, peridutal and common hepatic artery). Skeletonization of hepatic artery, portal vein and bile duct are recommended. Distant lymph node disease, such as coeliac, superior mesenteric and para-aortic, should be considered as M1 disease, and retrieval of these lymph nodes is not associated with improved survival. (Agreement 97.1%)*

### Question 13 When to resect the bile duct?

During a second operation, after a prior cholecystectomy the status of the cystic duct margin is of utmost importance. If positive for malignant cells, resection of the common bile duct, to optimize surgically negative margins with Roux-en-Y hepaticojejunostomy is recommended. Some authors suggest bile duct resection if the cystic duct stump cannot be identified and in selected young patients with biliopancreatic maljunction. In gallbladder cancers with the presence of perineural invasion, extra-hepatic bile duct resection presents significantly better survival than those without extra-hepatic bile duct resection^12^ ^,^ ^18^ ^,^ ^27^. The main finding of this review is that extra-hepatic bile duct resection is not preventative of loco-regional recurrence but can be curative in selected cases. Radical cholecystectomy with extra-hepatic bile duct resection is useful as a standard operation for tumors involving (macroscopically or microscopically) the neck and/or the cystic duct of the GB. In all other cases, the ability to achieve R0 resection, the presence of distant metastases, and extent of extra-hepatic bile duct resection, lymph node status and postoperative morbidity should guide the operative strategy^12^ ^,^ ^18^ ^,^ ^27^. A recent Japanese Society of Biliary Surgery survey reported that there was no benefit in overall survival in patients who underwent a routine bile duct resection. They concluded that extra-hepatic bile duct resection might be unnecessary in advanced gallbladder cancer without a direct infiltration of the hepatoduodenal ligament and the cystic duct. In gallbladder cancer resection, most of recurrences are distant metastasis and not local recurrence^12^ ^,^ ^18^ ^,^ ^27^. The potential adverse effect of extra-hepatic bile duct resection is the bilioenteric anastomosis that is associated with longer operation time and postoperative hospital stay, besides more blood transfusion. These factors are associated with significantly higher occurrence of postoperative complications^12^ ^,^ ^18^ ^,^ ^27^.

#### Consensus statement

*Common bile duct resection is indicated only in cases where is necessary to clear a positive cystic duct margin at the time of the original resection, in gallbladder cancer with direct infiltration of the hepatoduodenal ligament, in selected young patients with biliopancreatic maljunction, and in cases with intense postoperative fibrosis with significant hepatoduodenal ligament lymphadenopathy to facilitate adequate lymphadenectomy. Routine common bile duct resection is not indicated nor recommended, as it increases postoperative morbidity, does not increase the number of lymph nodes removed, and is not associated with overall survival improvement. (Agreement 97.1%)*

### Question 14 Is minimally invasive radical approach for gallbladder cancer feasible?

Minimally invasive approach for gallbladder cancer involves several complex procedures, such as hepatectomy, hepatoduodenal lymphadenectomy, and bilioenteric anastomosis. Beside the technical feasibility, the other component of a laparoscopic radical cholecystectomy is whether the long-term oncological outcomes are similar to that of an open procedure. Laparoscopic lymphadenectomy might yield a similar lymph node count compared with that of the open approach. A better magnification in minimally invasive approach will lead to adequate lymphadenectomy in order to obtain an R0 resection. Laparoscopic or robotic approach can be performed in patients with T1b-T3 tumors with liver and biliary tract involvement^5^ ^,^ ^15^ ^,^ ^22^ ^,^ ^35^. Wedge gallbladder bed resection, hepatic resection of segments IVb and V, and even extended resection cannot be considered a real limitation for the minimally invasive approach. In contrast, to perform bile duct resection and bilioenteric anastomosis, if necessary, may represent a technical difficulty^5^ ^,^ ^15^ ^,^ ^22^ ^,^ ^35^.

#### Consensus statement

*The current literature suggests that in specialized minimally invasive hepatopancreatobiliary centers laparoscopic or robotic radical cholecystectomy is safe and feasible with similar oncological outcomes compared to open approach. For laparoscopic or robotic approach, the expertise to perform lymphadenectomy, to achieve R0 resection, liver resection and bilioenteric anastomosis, are necessary. Multicenter, randomized controlled clinical trials are needed to objectively evaluate the clinical efficacy of minimally invasive approach for gallbladder cancer. (Agreement 97.1%)*

### Question 15 Routine port site excision is mandatory?

Port site recurrence is a major concern, and has been reported in 14-25% of patients with T2-T4 disease within 6-10 months. The possible mechanism responsible for port site recurrence include theories as direct contamination during specimen retrieval or contaminated instruments, contamination due to leakage of gas along the trocars, hematogenous dissemination, and changes in the host immune response^14^ ^,^ ^17^ ^,^ ^30^. It represents a disseminated disease that may not benefit from surgical resection. Port site resection was not associated with improved overall survival compared with no port site resection. The use of a plastic retrieval bag does not exclude the risk of disease recurrence at port sites. All patients with port site metastasis have T2 or T3 disease, and the majority of the patients have peritoneal carcinomatosis at the time of reoperation. Approximately 15% incidence of port site incisional hernia associated with port site resection was observed. Patients with disease recurrence at the port sites were identified as having residual disease at the time of reoperation^14^ ^,^ ^17^ ^,^ ^30^.^ ^ The one-year survival rate among patients with port site recurrence is less than 30%, and port site resection should not be routinely advocated during definitive surgical treatment. Port site metastasis is commonly associated with tumors in advanced stage, lymph node involvement and peritoneal carcinomatosis^14^ ^,^ ^17^ ^,^ ^30^. Isolated port site recurrence secondary to improper extraction technique is usually a localized recurrence. In patients with available information of extraction of gallbladder without bag and no disseminated disease, port site excision should be considered during re-resection^14^ ^,^ ^17^ ^,^ ^30^.

#### Consensus statement

*Port site resection is not associated with improved overall survival or lower distant disease recurrence. Most patients with disease recurrence at the port sites have residual disease at the time of reoperation associated with peritoneal carcinomatosis. Exceptionally, in cases with isolated port site recurrence due to improper extraction, without peritoneal disease, port site excision can be considered during re-resection. Port site resection is associated with higher incidence of incisional hernias. Routine port site resection is not recommended. (Agreement 97.1%)*

### Question 16 What is the role of adjuvant chemo (or chemoradiation) therapy?

So far the only regimen with overall survival benefit when compared to placebo in randomized phase III trials in resected biliary tract cancer is capecitabine based chemotherapy. In the BILCAP trial, only 18% of the patients had muscle-invasive gallbladder cancer. Although 38% had R1 disease, no patients received radiotherapy in the trial. Due to the lack of randomized trials, adjuvant chemoradiation has not been established as a standard of care. In the prospective single-arm SWOG0809 trial, patients with either resected extra-hepatic cholangiocarcinoma or gallbladder cancer received gemcitabine plus capecitabine followed by chemoradiation with capecitabine. Two-year survival rate was 56% in the gallbladder cancer group. However, the true role of adjuvant chemoradiotherapy for gallbladder cancer remains unknown. Nonetheless, patients with gallbladder cancer and microscopically positive surgical resection margin (R1/R2 resection margins) may be offered chemoradiation therapy following six months of adjuvant capecitabine^1^ ^,^ ^36^ ^,^ ^41^.

#### Consensus statement

*Adjuvant chemotherapy should be offered for patients with resected gallbladder cancer. For patients with it and a microscopically positive surgical margin (R1/R2 resection margins) chemoradiotherapy following adjuvant chemotherapy with capecitabine or adjuvant gemcitabine-based combination may be offered. (Agreement 97.1%)*

## CONCLUSIONS

It was possible to prepare safe recommendations as guidance for incidental gallbladder carcinoma, addressing the most frequent topics of everyday work of digestive and general surgeons.
